# Supplementary material for: Experience in Accessing Healthcare in Ethnic Minority Patients with Chronic Respiratory Diseases: A Qualitative Meta-Synthesis
Source: Healthcare (Basel). 2023 Dec 15;11(24):3170. doi: 10.3390/healthcare11243170 (PMC10743025; doi:10.3390/healthcare11243170)
Supplement: Supplementary file 1 [file healthcare-11-03170-s001.zip › healthcare-2728032-supplementary.pdf]

Supplementary Materials:

S1: Search keywords

**Lung Disease**	**Ethnicity**	**Healthcare service**	**Qualitative research**
respiratory disease OR lung disease	ethnic minority OR asian OR black OR african OR hispanic OR latin*	Health utility OR Healthcare service OR healthcare OR healthcare access OR healthcare disparity	Qualitative OR interview OR focus group OR phenom* OR grounded theory OR ethnography OR content analysis OR thematic analysis

*Asterisk used to retrieve different phrases that start with the same letters.

S2: Search databases

PubMed 21/04/2023		
Query	Search Term	Results
#1	((((respiratory disease) OR (lung disease)) AND ((((((Ethnic minority) OR (Asian)) OR (black)) OR (African)) OR (Hispanic)) OR (Latin*))) AND (((((health utility) OR (healthcare)) OR (healthcare access)) OR (healthcare service)) OR (healthcare disparity))) AND ((((((((Qualitative) OR (interview)) OR (focus group)) OR (phenom*)) OR (grounded theory)) OR (ethnography)) OR (content analysis)) OR (thematic analysis))	1,971
#2	((((respiratory disease) OR (lung disease)) AND (((((((Ethnic minority[Title/Abstract])) OR (Asian[Title/Abstract])) OR (black[Title/Abstract])) OR (African[Title/Abstract])) OR (Hispanic[Title/Abstract])) OR (Latin*[Title/Abstract]))) AND (((((health utility[Title/Abstract]) OR (healthcare[Title/Abstract])) OR (healthcare access[Title/Abstract])) OR (healthcare service[Title/Abstract])) OR (healthcare disparity[Title/Abstract]))) AND ((((((((Qualitative[Title/Abstract]) OR (interview[Title/Abstract])) OR (focus group[Title/Abstract])) OR (phenom*[Title/Abstract])) OR (grounded theory[Title/Abstract])) OR (ethnography[Title/Abstract])) OR (content analysis[Title/Abstract])) OR (thematic analysis[Title/Abstract]))	204

Scopus 21/04/2023		
Query	Search Term	Results
1	((((respiratory disease) OR (lung disease)) AND ((((((Ethnic minority) OR (Asian)) OR (black)) OR (African)) OR (Hispanic)) OR (Latin*))) AND (((((health utility) OR (healthcare)) OR (healthcare access)) OR (healthcare service)) OR (healthcare disparity))) AND ((((((((Qualitative) OR (interview)) OR (focus group)) OR (phenom*)) OR (grounded theory)) OR (ethnography)) OR (content analysis)) OR (thematic analysis))	112,670
2	((((respiratory disease[Title/Abstract]) OR (lung disease[Title/Abstract])) AND (((((((Ethnic minority[Title/Abstract])) OR (Asian[Title/Abstract])) OR (black[Title/Abstract])) OR (African[Title/Abstract])) OR (Hispanic[Title/Abstract])) OR (Latin*[Title/Abstract]))) AND (((((health utility[Title/Abstract]) OR (healthcare[Title/Abstract])) OR (healthcare access[Title/Abstract])) OR (healthcare service[Title/Abstract])) OR (healthcare disparity[Title/Abstract]))) AND ((((((((Qualitative[Title/Abstract]) OR (interview[Title/Abstract])) OR (focus group[Title/Abstract])) OR (phenom*[Title/Abstract]))	1

	OR (grounded theory[Title/Abstract])) OR (ethnography[Title/Abstract])) OR (content analysis[Title/Abstract])) OR (thematic analysis[Title/Abstract]))	

Web of Science 21/04/2023		
Query	Search Term	Results
1	(ALL=(respiratory disease)) OR ALL=(lung disease)	737, 905
2	(((((ALL=(ethnic minority)) OR ALL=(asian)) AND ALL=(black)) AND ALL=(african)) AND ALL=(hispanic)) AND ALL=(latin*)	541
3	((((ALL=(Health utility)) OR ALL=(Healthcare service)) OR ALL=(healthcare)) OR ALL=(healthcare access)) OR ALL=(healthcare disparity)	862, 741
4	(((((((ALL=(Qualitative)) OR ALL=(interview)) AND ALL=(focus group)) AND ALL=(phenom*)) AND ALL=(grounded theory)) AND ALL=(ethnography)) AND ALL=(content analysis)) AND ALL=(thematic analysis)	2
5	#4 AND #3 AND #2 AND #1	0
6	(TI=(respiratory disease)) OR TI=(lung disease)	48,169
7	(((((TI=(ethnic minority)) OR TI=(asian)) OR TI=(black)) OR TI=(african)) OR TI=(hispanic)) OR TI=(latin*)	523,300
8	(((((TI=(Health utility)) OR TI=(Healthcare service))) OR TI=(healthcare)) OR TI=(healthcare access)) OR TI=(healthcare disparity)	86,370
9	(((((((TI=(Qualitative)) OR TI=(interview)) OR TI=(focus group)) OR TI=(phenom*)) OR TI=(grounded theory)) OR TI=(ethnography)) OR TI=(content analysis)) OR TI=(thematic analysis)	333,378
10	#9 AND #8 AND #7 AND #6	0

CINAHL 22/04/2023		
Query	Search Term	Results
S1	TX respiratory disease OR TX lung disease	59,524
S2	TX ethnic minority OR TX asian OR TX black OR TX african OR TX hispanic OR TX latin*	249,991
S3	TX healthcare utility OR TX healthcare service OR TX healthcare OR TX healthcare access OR TX healthcare disparity	1,050,983
S4	TX qualitative OR TX interview OR TX focus group OR TX phenom* OR TX grounded theory OR TX ethnography OR TX content analysis OR TX thematic analysis	599,787
S5	S1 and S2 and S3 and S4	351
S6	TI respiratory disease OR TI lung disease	8,225
S7	TI ethnic minority OR TI asian OR TI black OR TI african OR TI hispanic OR TI latin*	64,310
S8	TI healthcare utility OR TI healthcare service OR TI healthcare OR TI healthcare access OR TI healthcare disparity	124,434
S9	TI qualitative OR TI interview OR TI focus group OR TI phenom* OR TI grounded theory OR TI ethnography OR TI content analysis OR TI thematic analysis	76,134
S10	S6 and S7 and S8 and S9	0

S3: Outcomes derived from included papers

1. Tumiel-Berhalter, L. and Zayas, L. E. (2006) 'Lay Experiences and Concerns with Asthma in an Urban Hispanic Community', Journal of the National Medical Association, 98(6), pp. 875–880. [33]

**Findings**	**Illustration from papers**	**Evidence**
Deceiving nature of asthma	'... I call it my enemy... the demon that comes after [me] ... When I'm in the middle of something good, this little devil comes out of nowhere and attacks me ... I can't stand it.' (Patient)	Unequivocal
Emergency department use	'...for insurance reasons, you don't have any medication at home' (Patient)	Unequivocal
Medication use and side effects	Patients were uncomfortable with unwanted reactions to prescriptions e.g. catarrh. (Apprehension of the investigator)	Credible

2. Poureslami, I. et al. (2011) 'Health Literacy, Language, and Ethnicity-Related Factors in Newcomer Asthma Patients to Canada: A Qualitative Study', Journal of Immigrant and Minority Health, 13(2), pp. 315–322. [25]

**Findings**	**Illustration from papers**	**Evidence**
Patients' Perceptions of Asthma: Concerns, Fears, and Hopes	'I am always worried that I would be able to do nothing about it' (Patient)<br><br>'I thought there was no hope to survive' (Patient)<br><br>'Afraid of getting addiction with the medication' (Patient)	Unequivocal
Lack of Cultural Sensitivity and Empathy	'Doctors don't have time to listen to you and explain things' (Patient)<br><br>'If we can have access to sources of our own language, we will be better able to get more and relevant information' (Patient)<br><br>'I changed my doctors because he/she didn't respect my culture' (Patient)<br><br>'I felt stereotyping and sort of attitudes by care providers and hospital staff' (Patient)	Unequivocal
Cultural, Language, and Systemic Barriers to Information and Care Services	'I would like to see that there is a health care system where newcomers who cannot speak English well still access to needed services and help' (Patient)<br><br>'When we first arrived in Canada we did not have the (sic) MSP card and it was the first barrier to services; I had to bring the medicine in my luggage' (Patient)	Unequivocal

Reliable Asthma Information: Trusted Sources	'I trust my doctor because he is a university professor' (Patient)	Unequivocal

3. Patel, M. R. et al. (2014) 'Experiences addressing health-related financial challenges with disease management among African American women with asthma', Journal of Asthma. Informa Healthcare, 51(5), pp. 467–473.[31]

**Findings**	**Illustration from papers**	**Evidence**
Acceptance of the status quo	'When I get into a financial situation, I sit right here on this couch, and I pray about it' (Patient)	Unequivocal
Planning ahead, stockpiling medicines and sharing medicines	'I know there was a gap between jobs where I had no coverage, but I knew it was coming. So, I found myself stockpiling medication'<br><br>'You know, sometimes my kids, because they have asthma, so I give them an inhaler, if I have too many inhalers - because my kids don't have insurance' (Patient)	Unequivocal
Utilizing community assistance programs	'There's a website you could go to, and it will pull up every single, pharmaceutical, every medication that you can click on, you know, it will take you to the company that makes the medication and whether or not they have a program' (Patient)	Unequivocal
Reaching out to healthcare providers and social networks	'Um, there have been times a doctor suggested something, and I saw, you know, I just, I don't think I can afford that at this time. So, let me see if there's another option for you' (Patient)	Unequivocal
Forgo self-management and seek urgent care	'Yeah, I've gone a year or two without taking medication all together just because I didn't have insurance' (Patient)	Unequivocal

4. Melton, C. et al. (2014) 'Health literacy and asthma management among African-American adults: an interpretative phenomenological analysis', Journal of Asthma. Informa Healthcare, 51(7), pp. 703–713.[27]

**Findings**	**Illustration from papers**	**Evidence**
Information desired versus information received	'No they never sent me to an educational class, but that probably would be good for someone like me that has the determination that they decide that I'm not gonna use that pump' (Patient)<br><br>'What is asthma? What medications that they have on the market for asthma. Uh, what uh triggers. What can trigger asthma? And what you need to do once you realize that you're having a asthma attack because I'm telling you, a lot of people don't know they havin' a asthma attack' (Patient)	Unequivocal

	'When I really learned about asthma, I learned it from going into the computer and pullin' stuff out.' (Patient)	
Trial and error	'I see that it's a trial and error which you can't really afford to have errors' (Patient)<br><br>'Um most of the time, I feel like I'm in control. But when you have a bad asthma attack, no you're not in control anymore' (Patient)	Unequivocal
Expectations of the patient-provider relationship	'I would just tell him [doctor]; I just want you to listen to me about what's going on with my body instead of writing while I'm talking to you' (Patient)<br><br>'You gonna talk to me or you gonna put me out, I'm gonna go find me another doctor' (Patient)<br><br>'You have to build rapport with your doctor' (Patient)<br><br>'I think when I first got pregnant and went to the doctor I was really timid.. you still had some of these prejudice white folks' (Patient)<br><br>'Uh pull up that coat and then he got a ink pen did something like he didn't want to touch me' (Patient)	Unequivocal

5. Lathan, C. S. et al. (2015) 'Perspectives of African Americans on Lung Cancer: A Qualitative Analysis', The Oncologist. Oxford University Press (OUP), 20(4), pp. 393–399.[29]

**Findings**	**Illustration from papers**	**Evidence**
Lung Cancer Knowledge	'Everybody I know that had contracted lung cancer, including our father, has died within 6 months' (Patient)<br><br>'The mortality rate is greater than other cancers' (Patient)	Unequivocal
Personal Risk for Lung Cancer	'I think anybody can have lung cancer with everything around them, so everybody's at risk' (Patient)<br><br>'That's so hard to answer because ... even though you have all these options, uh, what's the outcome on any of them? I mean, are you gonna live? Is there a chance you're gonna live? And what is the best treatment?' (Patient)	Unequivocal
Smoking Cessation	'I actually had a doctor say,"Oh, you just need to cut down a little. You don't need to stop smoking." But I think if I really felt like the doctors were concerned and that I had their support, and there was someone ... who was really sort of looking out for me, I would probably... make more of an attempt.' (Patient)	Unequivocal

Cause of Lung Cancer Disparities	‘I’ve lived in predominantly black communities, and I’ve lived in predominantly white neighborhoods, and I never see a 2-for-1 sale on Newports in white neighborhoods’ (Patient)	Unequivocal
Treatments for Lung Cancer	‘I have heard the treatment of chemotherapy. You get sicker faster’ (Patient)<br><br>‘Haven’t they got a better chance of missin’ some of the cancer … with surgery? And they got a better chance of gettin’ most of it with chemo?’ (Patient)	Unequivocal
Race and Quality of Care	‘I also think that there’s always been a disparity in terms of the medical treatment that black people get versus white people. I mean, you can literally walk downtown, and you see all these very health-looking white people walking down, and you see all these black people with, like, canes, and they’re, like, coughing, and their skin’s all screwed up.’ (Patient)	Unequivocal
Diagnosis Experience, Treatment, and Provider Trust	‘They knew what they were doin’, as far as I was concerned’ (Patient)	Unequivocal
Perceptions of What Causes Lung Cancer	‘I wished to heck that I had never smoked … that’s somethin’ that was in my control. I’m not too sure that my occupation, um, was in my control … but at that time, we never, nobody knew that asbestos did cause lung cancer’ (Patient)<br><br>‘It is probably viewed as one of those cancers that’s caused by something’ that you did, um, … but…when I say lung cancer, the first thought that probably went through somebody’s mind is that she was a smoker’ (Patient)	Unequivocal
Race and Quality of Care	‘I was a patient, and everybody just pitched right in and gave me the best advice and care’ (Patient)<br><br>‘I would think that it’s in the back of any African American’s ... mind ... I mean ... there’s certainly racism in ... in this country ... and yeah, things are changing but ... I always felt that there’s still racism ... in people’s actions’ (Patient)	Unequivocal

6. Lee, S. C. et al. (2016) ‘Elucidating patient-perceived role in “decision-making” among African Americans receiving lung cancer care through a county safety-net system’, Journal of Cancer Survivorship. Springer New York LLC, 10(1), pp. 153–163.[24]

**Findings**	**Illustration from papers**	**Evidence**
Challenges to Effective Communication	‘Well the doctor don’t tell me much…I have to bring it out of the doctor’ (Patient)	Unequivocal

	'They ain't saying nothing about nothing worsening, you know, getting bad or nothing... I believe if something was bad, they'll tell me' (Patient)	
Caregivers as Advocates and Facilitators	'I have to do the part that the doctors don't do and that gives me the motivation that okay, I know that hurt and I tell her that, 'oh, that was a bad one [radiation treatment],' but I don't stop. I'm not gonna stop because then…my taste buds coming back so little by little' (Patient)	Unequivocal
Lack of Understanding of Prognosis and Outcomes	'Is this ever gonna quit or if it ain't somebody needs to tell me' (Patient)<br><br>'From what he had told me earlier, it was like a phase 3 and I don't know what that is' (Patient)<br><br>'I was so worried about being cut though, I did not want to be cut at all, that I was grateful for because I didn't want to be cut…Because it's like every time somebody gets cut, it go like that *snap* and they say like the air hits and it spreads. I don't know if that's true or what but I do know that the majority of people that get cut, especially for lung cancer, they don't make it' (Patient)	Unequivocal
Clinicians make Decisions	'That's the number one thing it to understand what [doctors are] saying and if you do understand what they're saying, go about what they tell you to do. I mean if they - they're not gonna tell you to do anything wrong' (Patient)<br><br>'They always tell me what's going, you know, what they want me to know. I never really got the chance to just express out how I feel or what's going on' (Patient)	Unequivocal

7. Glasser, I. et al. (2016) 'Improving COPD Care in a Medically Underserved Primary Care Clinic: A Qualitative Study of Patient Perspectives', COPD: Journal of Chronic Obstructive Pulmonary Disease. Taylor and Francis Ltd, 13(5), pp. 616–621.[28]

**Findings**	**Illustration from papers**	**Evidence**
Issues related to quitting smoking	'Well, I've been smoking ever since I was - probably - 14 years old. When we worked at the tobacco farm, everybody started smoking there' (Patient)<br><br>'I told the doctor that I don't have the strength and the will power (to quit smoking) and he said: "I cannot deal with you. If you don't quit smoking, I will see you with an oxygen tank in one year."' (Patient)	Unequivocal

Problems dealing with second-hand smoke	'I had neighbors who smoked. I stayed on the third floor and they smoked on the steps- it came up to my apartment. There was nothing I could do about it' (Patient)	Unequivocal
Beliefs (myths) about quitting smoking	'when people quit smoking they get sicker' (Patient)<br><br>'Cigarettes do more harm to those who don't smoke than to those that do smoke' (Patient)	Unequivocal
Success in quitting smoking	'And when I decided "No, I not going to smoke anymore," they referred me to a woman here who was going to help me who is very nice. She saw me quickly and gave me two boxes of patches' (Patient)	Unequivocal
Difficulties paying for and obtaining medications	'I had a friend who used the same medicine and she used to give me some, which is not good but, it helped me because I couldn't afford it' (Patient)<br><br>'The problem is when they change the Medicaid or the insurance, that's it. One time they did not want to give me any more oxygen because the insurance did not want to cover it' (Patient)<br><br>'They gave me a medication that Medicaid doesn't pay for, so I just wait to get sick and go to the hospital' (Patient)	Unequivocal
Good experiences in obtaining and managing medications	'With CVS (a pharmacy) I don't have a problem, because when I bring in a prescription, the pharmacist asks me, "Do you want an automatic refill?" And I say yes' (Patient)<br><br>'Fortunately I'm blessed that my wife is a nurse and she takes care of my medication' (Patient)	Unequivocal
Difficulties utilizing machines	'The doctor told me that I have to use it (CPAP) but I can't use it every night because I can't sleep with it' (Patient)	Unequivocal
Challenge in receiving care from a teaching hospital clinic	'My suggestion for improvement is that they keep the doctors here because they change the doctors a lot here. When they change they come and ask the same questions' (Patient)<br><br>'My problem is that they change the doctors on me every one or two years' (Patient)	Unequivocal
Expressions of satisfaction	'My doctor treats me very well. My doctor is good' (Patient)<br><br>'I follow what my doctor says to do and he is the one that is right there for me' (Patient)	Unequivocal

8. Webb, L. A. and McDonnell, K. K. (2018) 'Not a Death Sentence: Perspectives of African American Women Living With Lung Cancer', Oncology Nursing Forum. Oncology Nursing Society, 45(1), pp. 46–54.[26]

Findings	Illustration from papers	Evidence
Judgement and lack of empathy from others (healthcare professionals, relatives, and peers)	'Oh my God. You're still alive? I can't believe it!' People expect me to look like I'm at death's door. They ask, 'Why do you look so good?'' (Patient)<br><br>'We already know there is stigma on our disease because they say it's the dirty disease, because we brought it on ourselves' (Patient)<br><br>'When you tell someone you have lung cancer, they are like, 'Did you smoke? Well, you brought this on yourself.'' (Patient)<br><br>'My doctor [did] not understand how hard it is to quit smoking. He never offered me help to quit' (Patient)	Unequivocal
Challenges with symptoms and unwanted consequences of treatment, with little assistance from doctors	'The doctor did not tell me once . . . that I was going to have scar tissue [from the radiation]' (Patient) 'They just give you a pamphlet and send you on you way', and also be less prejudice. For instance, 'Doctors think that we're not capable of comprehending what they're talking about' (Patient)	Unequivocal
Doctors not keeping patients informed	'Doctors think that we're not capable of comprehending what they're talking about' (Patient)	Unequivocal

9. Druedahl, L. et al. (2018) 'Young Muslim Women Living with Asthma in Denmark: A Link between Religion and Self-Efficacy', Pharmacy (Basel). MDPI AG, 6(3), p. 73.[30]

Findings	Illustration from papers	Evidence
Perceptions of Asthma and the Experience of Having Asthma	'If I usually use [terbutaline, relief medicine] and it doesn't help, it gets even worse' (Patient)	Unequivocal
Expectations about Medicine to Control Asthma Symptoms	'I don't have to do anything [to remember to use medicine]. When I don't use it, my coughing reminds me right away' (Patient)<br><br>'I don't think I see a difference in the one [controller] that I use morning and evening. But more to a higher degree with the one I use when necessary; its effect I feel right away' (Patient)<br><br>'I was told that it would go away when I used my inhaler daily for a while, but I haven't seen the effect yet' (Patient)	Unequivocal
Influence of Religion, Modes of Agency, and Ramadan on Self-Efficacy and Asthma Medicine Adherence	'according to my religion, Allah Teala [God] gives an illness to someone to cleanse their sins/ . . . /I see it [asthma] as an opportunity to cleanse my sins/. . . /He [God] loves me so much that he wants me in Paradise, so I have been blessed with an illness . . .' (Patient)	Unequivocal

	'…if you are only using the medicine once in the morning, then you shift it to somewhere in the evening. In that way, you are both fasting and complying with the medicine' (Patient)	

10. Ahmed, S. et al. (2022) 'Generational perspective on asthma self-management in the Bangladeshi and Pakistani community in the United Kingdom: A qualitative study', Health Expectations. John Wiley and Sons Inc, 25(5), pp. 2534–2547.[32]

**Findings**	**Illustration from papers**	**Evidence**
Self-management orientated through acculturation	'I feel 'erh, Bangladesh are more lucky. Bangladesh people. If you pay, you see a good doctor' (Patient)<br><br>'From the sweat, my cold comes, and my asthma happens, and I get breathless' (Patient)<br><br>'It's not as, because it's alien in the community a lot of things are alien and anything that's alien, is either frowned upon or ridiculed' (Patient)	Unequivocal
Self-management orientated through accultural stress	'I am in depression like you know I have, those things also because of my depression, stress, and this is the by-product of European environment., ' (Patients)<br><br>'There is a lot more stress than, 'erh non-Asians…[a white person] they don't deal with any of this rubbish. Yeah definitely, I think because they have a lot less things that they worry about and stress about' (Patients)	Unequivocal
Self-management orientated through social dialogues	'That's why I don't feel like it is talked about, because it's so just normal…' (Patient)<br><br>'I know a few of my friends, but them, they don't need the medicine every day like me…, but we don't talk about how we deal with it or anything' (Patient)	Unequivocal
Self-management orientated through medicalization	'Just don't see it [asthma self-management] as an issue really. Like if you got a headache you take paraceta-mol, you know' (Patient)<br><br>'50 years ago, there was, probably no treatment. So, people will have to live with it' (Patient)<br><br>'I don't really need this… The spacer… They shouldn't be making too much effort on generalisation as, 'You should do this'. Same thing does not fit everyone' (Patient)	Unequivocal

S4: Meta-synthesis

Findings	Groups	Synthesised findings
Judgement and lack of empathy from others (healthcare professionals, relatives, and peers) (U)	Self-awareness of being prejudiced	**Synthesised finding 1: The relationship between patients and healthcare professionals affects the usage of healthcare services.** Patients think that they need a positive connection with healthcare professionals where ethnic minorities can access the same care as majority groups for their condition to be well managed.
Lack of cultural sensitivity and empathy (U)		
Challenges to effective communication (U)		
Cultural, language, and systemic barriers to information and care services (U)		
Expectations of the patient-provider relationship (U)		
Race and quality of Care (U)		
Race and quality of Care (U)		
Lack of understanding of prognosis and outcomes (U)	Patients felt uninformed	
Clinicians make decisions (U)		
Information desired versus information received (U)		
Doctors not keeping patients informed (U)		
Challenges with symptoms and unwanted consequences of treatment, with little assistance from doctors (U)		
Challenge of receiving care from a teaching hospital clinic (U)	Reality of medical services	
Expressions of satisfaction (U)		
Reliable asthma information: trusted sources (U)		
Diagnosis experience, treatment, and provider trust (U)		
Good experiences in obtaining and managing medications (U)	Thoughts regarding treatment	**Synthesised finding 2: Patients' perceptions and cultural beliefs affect their compliance with disease management.** People of different backgrounds may have diverse thoughts about lung disease and experiences of interventions, leading to a particular viewpoint that impacts their willingness to engage with them.
Difficulties utilizing machines (U)		
Trial and error (U)		
Treatments for lung cancer (U)		
Patients' perceptions of asthma: concerns, fears, and hopes (U)		
Medication use and side effects (C)		
Influence of religion, modes of agency, and Ramadan on self-efficacy and asthma medicine adherence (U)		
Expectations about medicine to control asthma symptoms (U)		

Lung cancer knowledge (U)	Insight into lung illnesses	
Personal risk for lung cancer (U)		
Perceptions of what causes lung cancer (U)		
Perceptions of asthma and the experience of having asthma (U)		
Cause of lung cancer disparities (U)		
Planning ahead, stockpiling medicines, and sharing medicines (U)	Monetary issues	**Synthesised finding 3: Inequalities in accessing healthcare services.** Ethnic minorities may not be able to completely use the measures that assist in treating lung diseases.
Acceptance of the status quo (U)		
Reaching out to healthcare providers and social networks (U)		
Forgo self-management and seek urgent care (U)		
Difficulties paying for and obtaining medications (U)		
Emergency department use (U)		
Issues related to quitting smoking (U)	Smoking discontinuation	
Problems dealing with second-hand smoke (U)		
Beliefs (myths) about quitting smoking (U)		
Success in quitting smoking (U)		
Difficulty dealing with multiple illnesses (U)	Seeking alternative help	**Synthesised finding 4: Personal behaviours affect the usage of healthcare services.** Ethnic minorities may find it hard to regulate their lung disease.
Utilizing community assistance programs (U)		
Caregivers as advocate and facilitator (U)	Personal regulation of lung diseases	
Self-management orientated through medicalization (U)		
Speaking to patients in a similar position (U)		
Self-management orientated through social dialogues (U)		
Self-management orientated through acculturation (U)		
Self-management orientated through accultural stress (U)		

S5. ConQual summary of findings

Synthesised finding	Type of research	Dependability	Credibility	ConQual score
**Systematic review title:** Experience in accessing healthcare utilities by ethnic minority respiratory disease patients: a qualitative meta-synthesis.<br>**Population:** Ethnic minority groups with lung disease.<br>**Phenomena of interest:** Inequalities experienced by ethnic minorities when accessing healthcare services for lung disease.<br>**Context:** Qualitative investigations occurring across every healthcare venue involving ethnic minorities with lung disease.				
**Synthesised finding**	**Type of research**	**Dependability**	**Credibility**	**ConQual score**
**Synthesised finding 1: The relationship between patients and healthcare professionals affects the usage of healthcare services.**<br>Patients think that they need a positive connection with healthcare professionals where ethnic minorities can access the same care as majority groups for their condition to be well managed.	Qualitative	High	High	Moderate
**Synthesised finding 2: The way that perceptions of patients impact compliance with treatments for lung disease.**<br>People of different backgrounds may have diverse thoughts about lung disease and experiences of interventions, leading to a particular viewpoint that impacts their willingness to engage with them.	Qualitative	High	Downgrade 1 level**	Moderate
**Synthesised finding 3: Inequalities in accessing healthcare services.**<br>Ethnic minorities may not be able to completely use the measures that assist in treating lung diseases.	Qualitative	High	High	Moderate
**Synthesised finding 4: Personal behaviours affect the usage of healthcare services.**<br>Ethnic minorities may find it hard to regulate their lung disease.	Qualitative	High	High	Moderate

**The credibility downgrades one level due to a mix of unequivocal and credible findings (Munn et al., 2014).
